# BEARscc determines robustness of single-cell clusters using simulated technical replicates

**DOI:** 10.1038/s41467-018-03608-y

**Published:** 2018-03-22

**Authors:** D. T. Severson, R. P. Owen, M. J. White, X. Lu, B. Schuster-Böckler

**Affiliations:** 1 Ludwig Institute for Cancer Research, Nuffield Department of Clinical Medicine, University of Oxford, Oxford, OX3 7DQ UK; 2 Oxford University Hospital NHS Trust, John Radcliffe Hospital, Oxford, OX3 7DQ UK

## Abstract

Single-cell messenger RNA sequencing (scRNA-seq) has emerged as a powerful tool to study cellular heterogeneity within complex tissues. Subpopulations of cells with common gene expression profiles can be identified by applying unsupervised clustering algorithms. However, technical variance is a major confounding factor in scRNA-seq, not least because it is not possible to replicate measurements on the same cell. Here, we present BEARscc, a tool that uses RNA spike-in controls to simulate experiment-specific technical replicates. BEARscc works with a wide range of existing clustering algorithms to assess the robustness of clusters to technical variation. We demonstrate that the tool improves the unsupervised classification of cells and facilitates the biological interpretation of single-cell RNA-seq experiments.

## Abstract (web summary)

Single cell messenger RNAseq allows the study of heterogeneity in tissue samples. Here the authors present BEARscc, a tool that uses RNA spike-in controls to simulate experiment-specific technical replicates to estimate the robustness of computational predictions of subpopulations of cells.

## Introduction

The gene expression landscape of single cells can reveal important biological insights into the processes driving development or disease. The development of techniques to sequence mRNA from individualized cells (scRNA-seq) has enabled researchers to study cell subpopulations, including rare cell types, at an unprecedented scale and resolution^1–3^.

However, scRNA-seq has inherently high technical variability, and it is not possible to have true technical replicates for the same cell. This presents a major limitation for scRNA-seq analysis^4, 5^. Specifically, read count measurements often vary considerably as a result of stochastic sampling effects, arising from the limited amount of starting material^4, 5^. Also, false-negative observations frequently occur because expressed transcripts are not amplified during library preparation (the drop-out effect)^4, 5^. Another common problem is systematic variation due to minute changes in sample processing; these batch-dependent differences in cDNA conversion, library preparation and sequencing depth can easily mask biological differences among cells and might compromise many published scRNA-seq results^2, 6^.

One widely adopted approach to adjust for technical variation between samples is the addition of known quantities of RNA spike-ins to each cell sample before cDNA conversion and library preparation^7^. Several methods use spike-ins to normalize read counts per cell before further analysis^8, 9^, but this use has been criticized because it exacerbates the effect of differences in RNA content per cell, e.g., due to variations in cell size^2, 8^. Unfortunately, the limited volumes of starting material in single-cell transcriptomics inherently preclude the possibility of true technical replication.

To address this shortcoming of scRNA-seq analysis, we developed BEARscc (Bayesian ERCC Assessment of Robustness of single-cell clusters), an algorithm that uses spike-in measurements to model the distribution of experimental technical variation across samples to simulate realistic technical replicates. The simulated replicates can be used to quantitatively and qualitatively evaluate the effect of measurement variability and batch effects on analysis of any scRNA-seq experiment, facilitating biological interpretation. BEARscc represents a use for spike-in controls that is not subject to the same problems as per-sample normalization.

In many scRNA-seq studies, statistical clustering methods are used to identify cells with similar gene expression profiles that could represent distinct cell types^1, 10, 11^. BEARscc was designed specifically with this application in mind. The simulated technical replicates generated by BEARscc can be fed into most existing clustering algorithms. The BEARscc package provides analysis tools to evaluate the resulting replicate clusters, and can thus reveal how robust the classification of cells into subtypes is to technical variation.

## Results

### Outline of BEARscc workflow

Conceptually, BEARscc addresses the lack of experimental technical replicates in single-cell studies by simulating technical replicates. These simulated technical replicates are based on RNA spike-ins included in the experiment. Because RNA spike-ins have undergone the same sequencing steps as the cellular RNA, they can be used to create an experiment-specific model of the technical variability. The simulated replicates can then be analyzed using almost any existing clustering method (to group cells with similar gene expression profiles) as a way of assessing how technical variation might influence the clusters identified in the real experimental data (i.e., how ‘robust’ the clusters are to technical variation). This helps in the identification of clusters that are most likely to represent real biological sub-populations of cells.

**Fig. 1:**
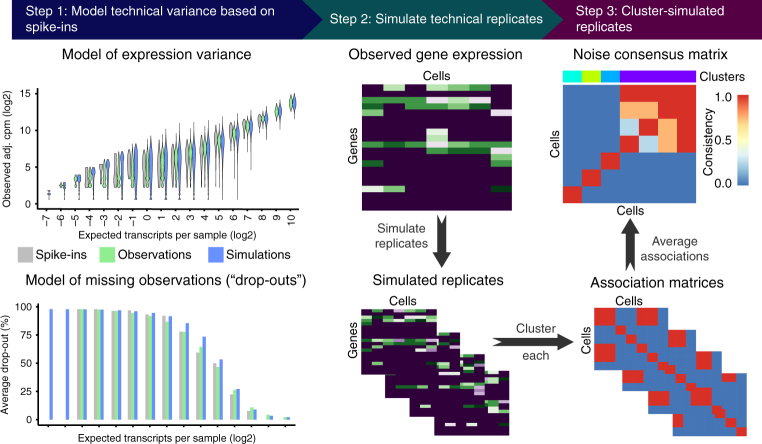
Overview of the BEARscc algorithm. Step 1, the variance of gene expression expected in a replicate experiment is estimated from the variation of spike-in measurements. Top: variation in spike-in read counts corresponds well with experimentally observed variability in biological transcripts (for details of control experiment see Methods) and read counts simulated by BEARscc. Bottom: drop-out likelihood is modelled separately, based on the drop-out rate for spike-ins of a given concentration. Shown is the average percentage drop-out rate as a function of the number of transcripts per sample, for spike-ins, simulated replicates and experimental observations in a control experiment (see Methods). Step 2, simulating technical replicates: the observed gene counts (top matrix) are transformed into multiple simulated technical replicates (bottom) by repeatedly applying the noise model derived in Step 1 to every cell in the matrix. Step 3, calculating a consensus: each simulated replicate (from Step 2) is clustered to create an association matrix. All the association matrices (bottom) are averaged into a single noise consensus matrix (top) that reflects the frequency with which cells are observed in the same cluster across all simulated replicates. Based on this matrix, noise consensus clusters can then be derived (coloured bar above matrix)

BEARscc consists of three steps (Fig. 1): modelling technical variance based on spike-ins (Step 1); simulating technical replicates (Step 2); and clustering simulated replicates (Step 3). In Step 1, an experiment-specific model of technical variability (noise) is estimated using observed spike-in read counts. This model consists of two parts. In the first part, expression-dependent variance (i.e., the expected variance of expression levels for a gene with a particular abundance) is approximated by fitting read counts of each spike-in across cells to a mixture model (see Methods). The second part addresses drop-out effects (i.e., false-negative observations that occur if expressed transcripts are not amplified during library preparation). Based on the observed drop-out rate for spike-ins of a given concentration, BEARscc generates a ‘drop-out injection distribution’, which models the likelihood that a given transcript concentration will result in a drop-out. Next, a ‘drop-out recovery distribution’ is estimated from the drop-out injection distribution using Bayes’ theorem; the drop-out recovery distribution models the likelihood that a transcript that had no observed counts in a cell was a false negative.

In Step 2, BEARscc applies the model from Step 1 to produce simulated technical replicates. For every observed gene’s read count (in the real experimental data set) below which drop-outs occurred amongst the spike-ins, BEARscc assesses whether to convert the count to zero (using the drop-out injection distribution). For observations where the read count is zero, the drop-out recovery distribution is used to estimate a new value, based on the overall drop-out frequency for that gene. After this drop-out processing, all non-zero read counts are substituted with a value generated by the model of expression-dependent variance (from the first part of Step 1), parameterized to the observed counts for each gene. Step 2 can be repeated any number of times to generate a collection of simulated technical replicates.

This set of simulated technical replicates can then be re-analyzed in the same way as the original experimental observations to assess the robustness of the results to modelled technical variation. Specifically, in Step 3 we focus on clustering analysis as this is a common approach for analyzing scRNA-seq data to identify groups of cells with similar gene expression profiles. Each simulated technical replicate is clustered using the same algorithm parameters as the original experimental observations. An association matrix is created in which each element indicates whether two cells share a cluster identity (1) or cluster apart from each other (0) in a particular replicate (Fig. 1, step 3). We provide a visual representation of the clustering variation on a cell-by-cell level by combining association matrices to form the ‘noise consensus matrix’. Each element of this matrix represents the fraction of simulated technical replicates in which two cells cluster together (the ‘association frequency’), after using a chosen clustering method.

To quantitatively evaluate the results, three metrics are calculated from the noise consensus matrix. “Stability” is the average frequency with which cells within a cluster associate with each other across simulated replicates. A stability value above 0.5 indicates that more cells than expected by chance are grouped together irrespective of technical variance. ‘Promiscuity’ measures the association frequency between cells within a cluster and those outside of it. A promiscuity value above 0.5 signifies that some cells in the cluster are better placed in other clusters. ‘Score’ is the difference between stability and promiscuity and reflects the overall robustness of a cluster to technical variance. A value above 0 suggests that the grouping of cells in this cluster is not purely an artefact of technical variance (Supplementary Fig. 1).

Determining the optimal number of clusters, *k*, into which cells should be grouped is an inherently difficult problem in scRNA-seq analysis. Heuristics, such as the silhouette index or the gap statistic^12, 13^, are commonly used (e.g., in RaceID2). Other tools, such as BackSPIN, employ custom algorithms to arrive at a fixed number of *k*, while e.g., SC3 leaves the decision to the user. All of these approaches, however, fail to account for the expected technical variance between measurements of single cells. BEARscc’s score statistic can help to refine what *k* to use, given a clustering algorithm. By performing hierarchical clustering on the noise consensus matrix, BEARscc can split cells into any number of clusters between 1 and *N* (the total number of cells). The hierarchical clustering with a maximum score (within a biologically reasonable range) represents a ‘meta-clustering’ with an optimal trade-off between within-cluster stability and between-cluster variability (see Methods). This meta-clustering methodology enables a semi-automatic refinement of existing clustering results.

### Evaluation of the BEARscc model of technical variance

Given the difficulty of generating true technical replicates from single-cell material, we generated a set of experimental replicates for which we diluted one RNA-seq library derived from bulk human brain tissue to single-cell RNA concentrations and sequenced 48 of these samples with ERCC spike-ins^12^. Each of these 48 samples is a ‘real’ technical replicate to compare to the simulated technical replicates generated by BEARscc. The mean and variance of the simulated read counts produced by BEARscc closely matched the experimentally determined values (Fig. 1, step 1—top; Supplementary Fig. 2a, b). For 95% of the genes expressed in the library, the simulated drop-out rate differed from the observed drop-out rate by <9% (Fig. 1, step 1—bottom; Supplementary Fig. 2c). Together, these results suggest that technical variation simulated by BEARscc closely resembles technical variation observed experimentally. The simulated expression of genes with less than 1 observed count deviated slightly from the experimentally determined values (Supplementary Fig. 2a), however such small expression differences are unlikely to be reproducible as they fall outside the dynamic range of any single cell experiment.

### Testing the utility of BEARscc in clustering analysis

**Fig. 2:**
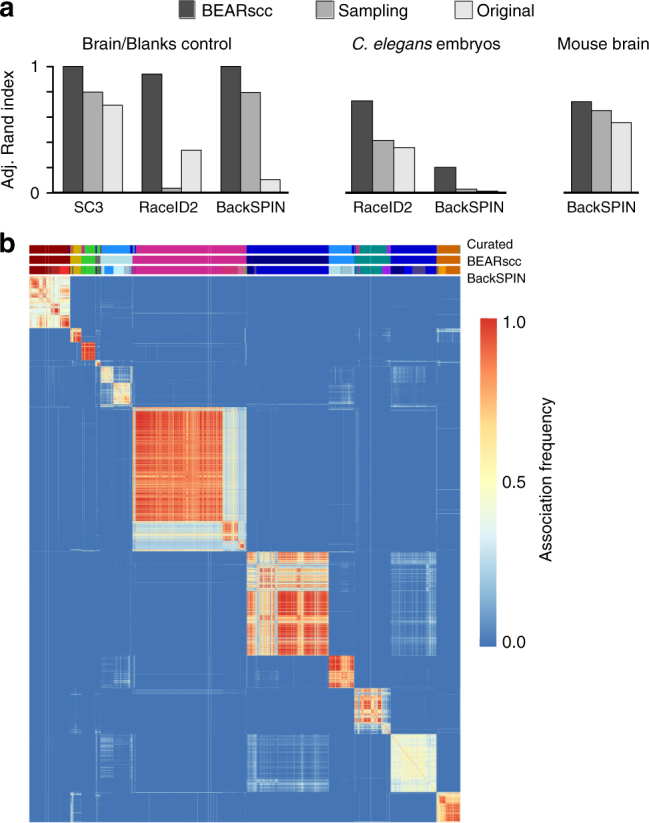
BEARscc improves clustering results and aids the interpretation of biological results. **a** Comparison of clustering accuracy of control data (left), *C*. *elegans* data (middle), and murine brain data (right). Adjusted Rand index denotes agreement with the manually annotated grouping of samples (1: perfect, 0: no overlap). ‘BEARscc’ indicates that BEARscc was used to generate simulated technical replicates that were clustered using the algorithm indicated below the graph; ‘Sampling’ indicates that a sub-sampling approach (see text) was used before clustering with each algorithm; ‘Original’ indicates that the clustering algorithm was used alone. **b** Example of a noise consensus matrix produced by BEARscc on data from murine brain cells (from Zeisel et al.^11^) clustered with BackSPIN. Bars above heatmap show the manually curated clustering of cells (top), BEARscc consensus cluster (middle) and unsupervised BackSPIN clusters (bottom)

To test whether BEARscc can improve the detection of true subpopulations of cells from single-cell transcriptome analysis, we performed a control experiment in which we sequenced 45 ‘blank’ samples opposite the diluted brain RNA, in two batches. The blanks only contained spike-ins and trace amounts of environmental contamination, producing sporadic read counts. We clustered the data from the brain samples and blanks using three widely used clustering algorithms (RaceID2^10^, BackSPIN^11^, and SC3^14^), either alone or after simulating technical replicates using BEARscc. Correct clustering should give perfect separation of brain and blank samples. To avoid artifacts due to differences in amplification-dependent library size, we applied an adjusted cpm normalization (see Methods). Otherwise, standard parameters were used for all three clustering algorithms. As an alternative to BEARscc, we also tested a simple sampling approach where we repeatedly sampled half of all expressed genes and re-clustered the cells based on this subset (see Methods). Without BEARscc or this sampling approach, all three clustering algorithms created false-positive clusters (Fig. 2a, Supplementary Fig. 3a-c, top). BEARscc provided a clear improvement over the original clustering and the sampling approach (Fig. 2a). Overall, BEARscc separated brain tissue and blank samples correctly and eliminated spurious clusters that corresponded to batch effects (Supplementary Fig. 3a, c, coloured bars above matrices). In the case of using BEARscc with RaceID2, three outlier cells were incorrectly identified to be robust clusters (Supplementary Fig. 3b, coloured bars above matrix); the libraries for these three samples contained fewer than 1000 observed transcripts, indicating that BEARscc is limited by RaceID2’s oversensitivity to library size differences.

### Applying BEARscc to a well annotated biological data set

To further test the utility of BEARscc, we applied BEARscc to a previously published data set^15^ of scRNA-seq measurements from early *C. elegans* embryogenesis. In this study, mRNA from four biological replicates of cells from the 1, 2, 4, 8 and 16-cell stage was sequenced. We made the assumption that cells from the 1, 4 and 16-cell stages are more different between than within stages. We therefore expected to observe clusters that preferentially group cells from the same stage across biological replicates. To test this, we ran RaceID2^10^ and BackSPIN^11^ on the expression data from these three distinct stages and compared the resulting cluster assignments to the meta-clusters created by BEARscc, or by gene sampling as described above. We found that BEARscc outperformed gene sampling and either RaceID2 or BackSPIN alone, as measured by the adjusted rand index (expecting three distinct clusters, see Fig. 2a). RaceID2 alone produced 10 clusters, which were reduced to two by BEARscc (Supplementary Fig. 4a and Supplementary Fig. 5a, b). BackSPIN alone resulted in 34 clusters, which BEARscc merged to 7 clusters (Supplementary Fig. 4b and Supplementary Fig. 5c, d). These data suggest that BEARscc reduces over-clustering by commonly used clustering algorithms and improves the interpretation of scRNA-seq data.

### BEARscc enhances the interpretation of published data sets

To assess the robustness of computational cell-type detection based on real biological scRNA-seq data, we applied BEARscc to two previously published data sets. We first re-analyzed murine brain data (3005 cells) from Zeisel et al.^11^, using BEARscc together with BackSPIN, which is the clustering algorithm used in the original publication. Based on the score statistic, BEARscc reduced the 24 clusters produced by BackSPIN alone into 11 clusters, which corresponded well with the manually curated cell types described in the original publication (Adjusted Rand Index 0.72 with BEARscc, and 0.55 for BackSPIN alone; Fig. 2a (right), Fig. 2b). Therefore, BEARscc provided an optimal grouping of cells without the effort of manual curation.

In a second evaluation, we re-analyzed murine intestinal data (291 cells) from Grün et al.^10^, using BEARscc to generate simulated technical replicates and RaceID2 (as described in the original publication) for clustering. The score metric from BEARscc indicated that 219 out of 291 cells were robustly classified in the original work. However, the two largest clusters—‘cluster 1’ and ‘cluster 2’—exhibited low scores (−0.07 and 0.20, respectively) compared to the other non-outlier clusters 3, 4 and 5 (Supplementary Fig. 6a). The BEARscc noise consensus matrix reveals high variability in the clustering patterns of cells in clusters 1 and 2 (Supplementary Fig. 6b). Grün et al. suggest that clusters 1 and 2 reflect closely related, undifferentiated cell types (‘transit-amplifying’ and ‘stem-like’, respectively). Expression patterns of genes characteristic of the two clusters were highly similar (Supplementary Fig. 7a), compared to the expression differences between cluster 1 and the next-largest cluster (cluster 5) (Supplementary Fig. 7b). Expression fold-changes between clusters 1 and 2 were reduced in technical replicates, falling below the significance threshold for many genes. BEARscc shows that many cells in clusters 1 and 2 cannot be reliably classified into one cluster or the other. The sharp distinction between clusters 1 and 2 described in the original publication is therefore likely to be a result of technical variation, rather than a defining biological feature of these cells. Instead, cells in clusters 1 and 2 seem to lie on a gradient of differentiation between two cellular states. More work will be needed to fully determine how the differentiation state of stem-like cells is reflected by their transcriptome. Nevertheless, this example demonstrates how BEARscc can help to improve the biological interpretation of scRNA data.

### Performance of BEARscc

With single-cell experiments becoming larger and larger, program execution time can become a bottleneck for computational analyses. Using simulated data, we show that BEARscc’s run-time grows linearly with increasing numbers of cells, assuming a constant number of genes (Supplementary Fig. 8). On a desktop PC (Intel i7 with 2.9 GHz), a single BEARscc process required ~16 min and 19 min to generate a simulated technical replicate from the *C. elegans* (14,448 genes by 115 samples) and murine brain (19,972 genes by 3005 samples) data, respectively. Importantly, the generation of simulated replicates can be distributed across multiple independent processes on multiple machines. The real time requirement for generating a replicate data sets is thus mostly limited by the available hardware. Once simulated technical replicates have been generated, the runtime of any downstream clustering analysis is dependent on the specifics of the respective clustering algorithm.

## Discussion

BEARscc addresses the challenges posed by intrinsically high technical variability in single-cell transcriptome sequencing experiments and enables the evaluation of single cell clustering results. Importantly, BEARscc is not a clustering algorithm in itself, but rather a tool to evaluate the results produced by any available clustering algorithm. To do so, it aggregates the information from exogenous control spike-ins across samples to create a model of both the expected variance of endogenous read counts and the likelihood of false-negative measurements (drop-outs). This represents an alternative use of spike-in controls that is not subject to potential issues surrounding the use of spike-ins for per-sample normalisation. Our application of BEARscc to biological datasets demonstrates that BEARscc reduces over-clustering, is able to identify biologically relevant cell groups in an unsupervised way and provides additional insights for the interpretation of single-cell sequencing experiments.

We note that extreme batch effects with a multi-modal distribution of variance could skew BEARscc’s noise model and lead to biased simulated replicates. We envision that future versions of BEARscc will attempt to detect and warn about such biases. Furthermore, while the drop-out model calculated by BEARscc is accurate for genes with an average expression of more than one count, there is still scope for improvement. Future work will focus on more precise models of drop-outs in the context of very low gene expression. As it stands, BEARscc enables users to identify the components of scRNA-seq clustering results that are robust to noise, thereby increasing confidence in those results for downstream analysis. Therefore, we recommend that future scRNA-seq analysis pipelines apply the best available clustering algorithm in conjunction with BEARscc in order to define the most biologically meaningful groups of cells for interpretation.

## Methods

### Public data analysis

Primary murine cortex and hippocampus single-cell measurements for 3005 cells from Zeisel et al.^11^ were retrieved from the publicly available Linnarsson laboratory data repository [http://linnarssonlab.org/cortex/]. Primary murine intestinal single cell measurements of 260 cells from Grün et al.^10^ were downloaded from the van Oudenaarden github repository [https://github.com/dgrun/StemID]. Primary *C. elegans* embryo single-cell measurements for 219 cells from Tintori et al.^15^ were obtained from the SRA^16^ sequencing database with project id SRP070155. Raw reads were mapped to PRJNA13758.WS259 using STAR^17^. Exact position duplicates were removed. These samples were deeply sequenced (~2 million reads or more per cell) resulting in saturated ERCC spike-ins with limited spike-in drop-outs. To allow BEARscc to build the noise models from spike-in data for simulating of technical replicates, mapped libraries were down-sampled to 20% of mapped reads. Features were counted using HTseq^17^. Samples identified by Tintori et al. as failing quality control were excluded, and counts were normalized to adjusted counts per million as described below.

### Algorithmic generation of simulated technical replicates

Simulated technical replicates were generated from the noise mixture model and two drop-out models. For each gene, the count value of each sample is systematically transformed using the mixture model, *Z*(*c*), and the drop-out injection, Pr(*X* = 0|*Y* = *k*), and recovery, Pr(*Y*_*j*_ = *y*|*X*_*j*_ = 0), distributions in order to generate simulated technical replicates as indicated by the following pseudocode:

FOR EACH gene, *j*

FOR EACH count, *c*

IF *c*=0

*n* ← SAMPLE one count,*y*, from Pr(*Y*_*j*_ = *y*|*X*_*j*_ = 0)

IF *n*=0

*c* ← 0

ELSE

*c* ← SAMPLE one count from *Z*(*n*)

ENDIF

ELSE

IF *c* ≤ *k*

dropout ← TRUE with probability, Pr(*X* = 0|*Y* = *k*)

IF dropout=TRUE

*c* ← 0

ELSE

*c* ← SAMPLE one count from *Z*(*c*)

ENDIF

ELSE

*c* ← SAMPLE one count from *Z*(*c*)

ENDIF

ENDIF

RETURN *c*

DONE

DONE

### Modelling noise from spike-ins

Technical variance was modelled by fitting a single parameter mixture model, *Z*(*c*) to th spike-ins’ observed count distributions. The noise model was fit independently for each spike-in transcript and subsequently regressed onto spike-in mean expression to define a generalized noise model. This was accomplished in three steps:

1. Define a mixture model composed of Poisson and Negative Binomial random variables: *Z*~(1 − *α*)*Pois(*μ*) + α*NBin(*μ*,*σ*)
2. Empirically fit the parameter, *α*_*i*_, in a spike-in-specific mixture-model, *Z*_*i*_, to the observed distribution of counts for each ERCC spike-in transcript, *i*, where *μ*_*i*_ and *σ*_*i*_ are the observed mean and variance of the given spike-in. The parameter, *α*_*i*_, was chosen such that the error between the observed and mixture model was minimized.
3. Generalize the mixture model by regressing *α*_*i*_ parameters and the observed variance *σ*_*i*_ onto the observed spike-in mean expression, *μ*_*i*_. Thus the mixture model describing the noise observed in ERCC transcripts was defined solely by *μ*, which was treated as the count transformation parameter, *c*, in the generation of simulated technical replicates.

In step 2, a mixture model distribution is defined for each spike-in, *i*: *Z*_*i*_(*α*_*i*_,*μ*_*i*_,*σ*_*i*_)~(1 − *α*_*i*_)*Pois(*μ*_*i*_) + *α*_i_*NBin(*μ*_*i*_,*σ*_*i*_). The distribution, *Z*_*i*_, is fit to the observed counts of the respective spike-in, where *α*_*i*_ is an empirically fitted parameter, such that the *α*_*i*_ minimizes the difference between the observed count distribution of the spike-in and the respective fitted model, *Z*_*i*_. Specifically, for each spike-in transcript, *μ*_*i*_ and *σ*_*i*_ were taken to be the mean and standard deviation, respectively, of the observed counts for spike-in transcript, *i*. Then, *α*_*i*_ was computed by empirical parameter optimization; *α*_*i*_ was taken to be the *α*_*i,j*_ in the mixture-model, *Z*_*i*,*j*_(*α*_*i*,*j*_,*μ*_*i*_,*σ*_*i*_)~(1 − *α*_*i*,*j*_)*Pois(*μ*_*i*_) + *α*_*i*,j_*NBin(*μ*_*i*_,*σ*_*i*_), found to have the least absolute total difference between the observed count density and the density of the fitted model, *Z*_*i*_. In the case of ties, the minimum *α*_*i*,*j*_ was chosen.

In step 3, *α*(*c*) was then defined with a linear fit, *α*_*i*_ = a*log2(*μ*_*i*_) + *b*. *σ*(*c*) was similarly defined, log2(*σ*_*i*_) = a*log2(*μ*_*i*_) + *b*. In this way, the observed distribution of counts in spike-in transcripts defined the single parameter mixture model, *Z*(*c*), used to transform counts during generation of simulated technical replicates:

$$Z(c)\sim \left( {1 - \alpha \left( c \right)} \right) \ast {\mathrm{Pois}}\left( c \right) + \alpha (c) \ast {\mathrm{NBin}}\left( {c,\sigma (c)} \right).$$

During technical replicate simulation, the parameter *c* was set to the observed count value, *a*, and the transformed count in the simulated replicate was determined by sampling a single value from *Z*(*c=a*).

### Inference of drop-out distributions using spike-ins

A model of the drop-outs was developed to inform the permutation of zeros during noise injection. The observed zeros in spike-in transcripts as a function of actual transcript concentration and Bayes’ theorem were used to define two models: the ‘drop-out injection distribution’ and the ‘drop-out recovery distribution’.

The drop-out injection distribution was described by Pr(*X* = 0|*Y* = *y*), where *X* is the distribution of observed counts and *Y* is the distribution of actual transcript counts; the density was computed by regressing the fraction of zeros observed in each sample, *D*_*i*_, for a given spike-in, *i*, onto the expected number of spike-in molecules in the sample, *y*_*i*_, e.g., *D* = a*y + *b*. Then, *D* describes the density of zero-observations conditioned on actual transcript number, *y*, or Pr(*X* = 0|*Y* = *y*). Notably, each gene was treated with an identical density distribution for drop-out injection.

In contrast, the density of the drop-out recovery distribution, Pr(*Y*_*j*_ = *y*|*X*_*j*_ = 0), is specific to each gene, *j*, where *X*_*j*_ is the distribution of the observed counts and *Y*_*j*_ is the distribution of actual transcript counts for a given gene. The gene-specific drop-out recovery distribution was inferred from drop-out injection distribution using Bayes’ theorem and a prior. This was accomplished in 3 steps:

1. For the purpose of applying Bayes’ theorem, the gene-specific distribution, Pr(*X*_*j*_ = 0|*Y*_*j*_ = *y*), was taken to be the the drop-out injection density for all genes, *j*.
2. The probability that a specific transcript count was present in the sample, Pr(*Y*_*j*_ = *y*), was a necessary, but empirically unknowable prior. Therefore, the prior was defined using the law of total probability, an assumption of uniformity, and the probability that a zero was observed in a given gene, Pr(*X*_*j*_ = 0). The probability, Pr(*X*_*j*_ = 0), was taken to be the fraction of observations that were zero for a given gene. This was done to better inform the density estimation of the gene-specific drop-out recovery distribution.
3. The drop-out recovery distribution density was then computed by applying Bayes’ theorem:

1 $${\mathrm{Pr}}\left( {Y_j = y{\mathrm{|}}X_j = 0} \right) = \frac{{{\mathrm{Pr}}\left( {X_j = 0{\mathrm{|}}Y_j = y} \right) \ast {\mathrm{Pr}}\left( {Y_j = y} \right)}}{{{\mathrm{Pr}}\left( {X_j = 0} \right)}}.$$

In the second step, the law of total probability, an assumption of uniformity, and the fraction of zero observations in a given gene were leveraged to define the prior, Pr(*Y*_*j*_ = *y*). First, a threshold of expected number of transcripts, *k* in *Y*, was chosen such that *k* was the maximum value for which the drop-out injection density was non-zero. Next, uniformity was assumed for all expected number of transcript values, *y* greater than zero and less than or equal to *k*; that is Pr(*Y*_*j*_ = *y*) was defined to be some constant probability, *n*. Furthermore, Pr(*Y*_*j*_ = *y*) was defined to be 0 for all *y*>*k*. To inform Pr(*Y*_*j*_ = *y*) empirically, Pr(*Y*_*j*_ = 0) and *n* were derived by imposing the law of total probability (2) and unity (3) yielding a system of equations:

2 $$\Pr \left( {X_j = 0} \right) = \mathop {\sum }\limits_{y = 0}^k \left\{ {{\mathrm{Pr}}\left( {X_j = 0{\mathrm{|}}Y_j = y} \right) \ast {\mathrm{Pr}}\left( {Y_j = y} \right)} \right\}$$

3 $$\mathop {\sum }\limits_{y = 0}^k \left\{ {{\mathrm{Pr}}\left( {Y_j = y} \right)} \right\} = \Pr \left( {Y_j = 0} \right) + k \ast n = 1$$

The probability that a zero is observed given there are no transcripts in the sample, Pr(*X*_*j*_ = 0|*Y*_*j*_ = 0), was assumed to be 1. With the preceding assumption, solving for Pr(*Y*_*j*_ = 0) and *n* gives:

4 $$n = \frac{{1 - \Pr \left( {Y_j = 0} \right)}}{k}$$

5 $$\Pr \left( {Y_j = 0} \right) = \frac{{\Pr \left( {X_j = 0} \right) - \frac{1}{k} \ast \mathop {\sum }\nolimits_{y = 1}^k \left\{ {{\mathrm{Pr}}\left( {X_j = 0{\mathrm{|}}Y_j = y} \right)} \right\}}}{{1 - \frac{1}{k} \ast \mathop {\sum }\nolimits_{y = 1}^k \left\{ {{\mathrm{Pr}}\left( {X_j = 0{\mathrm{|}}Y_j = y} \right)} \right\}}}$$

In this way, Pr(*Y*_*j*_ = *y*) was defined by Eq. (4) for *y* in *Y*_*j*_ less than or equal to *k* and greater than zero, and defined by Eq. (5) for *y* in *Y*_*j*_ equal to zero. For *y* in *Y*_*j*_ greater than *k*, the prior Pr(*Y*_*j*_ = *y*) was defined to be equal to zero.

In the third step, the previously computed prior, Pr(*Y*_*j*_ = *y*), the fraction of zero observations in a given gene, Pr(*X*_*j*_ = 0), and the drop-out injection distribution, Pr(*X*_*j*_ = 0|*Y*_*j*_ = *y*), were utilized to estimate with Bayes’ theorem the density of the drop-out recovery distribution, Pr(*Y*_*j*_ = *y*|*X*_*j*_ = 0). During the generation of simulated technical replicates for zero observations and count observations less than or equal to *k*, values were sampled from the drop-out recovery and injection distributions as described in the pseudocode of the algorithm.

### Observing real technical noise

Brain whole tissue total RNA (Agilent Technologies, cat. 540005) was diluted to 10 pg aliquots and added to 1 μL. cDNA conversion, library preparation, and sequencing were performed by the Wellcome Trust Center for Human Genomics Sequencing Core. Blank samples were identically prepared with nuclease free water. Samples were pipetted into 96-well plates and treated as single cells using Smartseq2 cDNA conversion as described by Picelli et al.^19^ with minor modifications. The library was prepared using Fludigm’s recommendations for Illumina NexteraXT at ¼ volume with minor modifcations, and sequenced on the Illumina HiSeq4000 platform. Raw reads were mapped to hg19 using STAR^17^. Exact position duplicates were removed, and features were counted using HTseq^17^. Counts were normalized to adjusted counts per million as described below.

### Normalization of counts

For the brain and blanks control experiment and *C. elegans* data, raw counts were normalized to adjusted counts per million to reduce batch effects: raw counts were divided by the total number of counts and multiplied by 10^6^. For each sample library, a detection rate was computed by dividing the number of genes with at least one observed count by the total number of observed genes across all libraries. A scaling factor was then computed for each sample library by dividing each library’s detection rate by the median detection rate, as initially suggested by Hicks et al.^20^.

### Clustering of counts data

BackSPIN, SC3 and RaceID2 were run according to algorithm-specific recommendations^10, 11, 14^. RaceID2 was allowed to identify cluster number under default parameters. For the brain and blanks control experiment and *C. elegans* data, RaceID2 was modified to skip normalization since scaled counts per million normalization had already been applied to the data set. The number of clusters, *k*, selected for SC3 clustering was determined empirically by selecting *k* with the optimal silhouette distribution across noise injected counts matrices.

### Computation of consensus matrix

Hundred simulated replicate matrices for *n* cells and *m* genes were clustered using the respective clustering algorithm (SC3, BackSPIN, RaceID2) as described above. Cluster labels were used to compute an *n* × *n* binary association matrix for each clustering. Each element of the association matrix represents a cell–cell interaction, where a value of 1 indicates that two cells share a cluster and a value of 0 indicates two cells do not share a cluster. An arithmetic mean was taken for each respective element across the resulting 100 association matrices to produce an *n* × *n* noise consensus matrix, where each element represents the fraction of noise injected counts matrices that, upon clustering, resulted in two cells sharing a cluster.

### Computation of BEARscc cluster metrics

To calculate cluster stability, the noise consensus matrix was subset to cells assigned to the cluster. The cluster stability was then calculated as the arithmetic mean of the upper triangle of the subset noise consensus matrix. To calculate cluster promiscuity, the rows of the noise consensus matrix were subset to cells assigned to the cluster and the columns are subset to the cells not assigned to the cluster. For clusters with as many or more cells assigned to them than not assigned, the promiscuity was defined as the arithmetic mean of the elements in the subset matrix. Otherwise, the columns were further subset to the same number of cells as were assigned to the cluster, where the cells outside of the cluster with the strongest mean association with cells inside the cluster are chosen. The promiscuity was defined as the arithmetic mean of the elements in this further subset matrix. Each cluster’s promiscuity was subtracted from its stability to calculate cluster score.

### Computation of BEARscc cell metrics

To calculate a cell’s stability, the arithmetic mean was taken of that cell’s association frequencies with other cell’s within the cluster. To calculate a cell’s promiscuity, there were two cases. For cells in clusters with as many or more cells assigned to them than not assigned, the promiscuity was the arithmetic mean of that cell’s association frequencies with all cells not assigned to the relevant cluster. For cells in clusters of size *n*, with fewer cells assigned to them than not assigned, the cell’s promiscuity was the arithmetic mean with the *n* cells not assigned to the cluster with the highest association frequencies. Each cell’s promiscuity was subtracted from its stability to calculate cell score.

### Estimating the background distribution of BEARscc metrics

To compute null distributions for the stability, promiscuity and score metrics, random clusters were generated with varying numbers of cells *m* in a data set, where each cell was assigned to an arbitrary reference cluster with size *n*. We then computed 100 random association matrices by taking each possible cell-to-cell association and assigning a 0 or 1 with equal probability, i.e., a cell was equally likely to associate or not with any cell. The noise consensus matrix was computed from these 100 random association matrices. From the noise consensus, the score, stability, and promiscuity metrics were calculated both per cell and per cluster. This computation was repeated 100 times for each set *m* and *n* parameters to describe the null distribution of BEARscc metrics at the cell and cluster level.

### Evaluation of BEARscc runtime

To estimate the run-time requirements of BEARscc, a random sampling of 2000 samples and 20,000 genes was taken from the brain whole tissue counts matrix with replacement. This large count matrix was subset to an increasing number of genes (63, 504, 700, 1008, 1800 and 2016) and cells (6, 12, 25, 36, 50, 72) to generate counts matrices with increasing numbers of elements. The BEARscc functions estimate_noiseparameters and simulate_replicates were run on each count matrix on a standard desktop PC (Intel i7 with 2.9 GHz). The microbenchmark R package was used to measure the execution time for each counts matrix subset.

### Estimation of cluster number *k*

To determine the cluster number, *k*, from the hierarchical clustering of the noise consensus, the resulting dendrogram was cut multiple times to form *N* clusterings with cluster numbers *k*=1 to *k*=*N* clusters. The average score metric was computed for each clustering, and *k* was chosen by taking the *k* with the maximum average score metric. Evaluating all possible *k* from 1 to the number of cells in the experiment is computationally expensive and unlikely to be biologically meaningful. In this work, *N* was capped at 0.1 times the number of cells in the experiment: *N*=10 for the brain and blanks control, *N*=30 for the murine intestine experiment, and *N*=300 for the murine brain data.

### Gene sampling

For comparison with BEARscc, 100 subsampling iteration matrices for *n* cells and *m* genes were generated by sampling one half of expressed genes and clustered using the respective clustering algorithm (SC3, BackSPIN, RaceID2). For each data set, genes were excluded with less than 25 total raw counts across all samples in the cohort. The remaining genes formed the sample space. In each subsampling iteration, one half of the genes were sampled without replacement, and their expression across cells was used as the counts matrix. Identically to the computation of the BEARscc noise consensus matrix, cluster labels were used to compute an *n* × *n* binary association matrix for each clustering, and an arithmetic mean was taken for each respective element across the resulting 100 association matrices to produce an *n* × *n* subsampling consensus matrix. Identically to BEARscc analysis, the BEARscc score metric was used to determine cluster number *k*, and the resulting cluster labels for each data set and algorithm were compared with BEARscc by computing the adjusted rand index for each with respect to the relevant ground truth.

### Code availability

BEARscc[10.18129/B9.bioc.BEARscc] is freely available as an R package through Bioconductor^18^.

### Data availability

The single cell RNA-sequencing counts from primary murine brain and intestine are available on the Linnarsson laboratory data repository [http://linnarssonlab.org/cortex/] and the van Oudenaarden laboratory github repository [https://github.com/dgrun/StemID], respectively. The single RNA-sequencing raw reads from primary *C. elegans* embryo are available on the SRA^16^ sequencing database through the GEO data repository with accession number GSE77944[https://www.ncbi.nlm.nih.gov/geo/query/acc.cgi?acc=GSE77944]. The raw reads counts for the brain and blanks experiment are available on the GEO data repository with accession number GSE95155 [https://www.ncbi.nlm.nih.gov/geo/query/acc.cgi?acc=GSE95155].

## Electronic supplementary material

Supplementary Information(PDF 1933 kb)
