# Supplementary material for: BEARscc determines robustness of single-cell clusters using simulated technical replicates
Source: Nat Commun. 2018 Mar 22;9:1187. doi: 10.1038/s41467-018-03608-y (PMC5864873; doi:10.1038/s41467-018-03608-y)
Supplement: Supplementary file 1 — Supplementary Information(PDF 1933 kb) [file 41467_2018_3608_MOESM1_ESM.pdf]

Supplementary Information

BEARscc determines robustness of single-cell clusters using simulated technical replicates

Severson *et al.*

Supplementary Figures

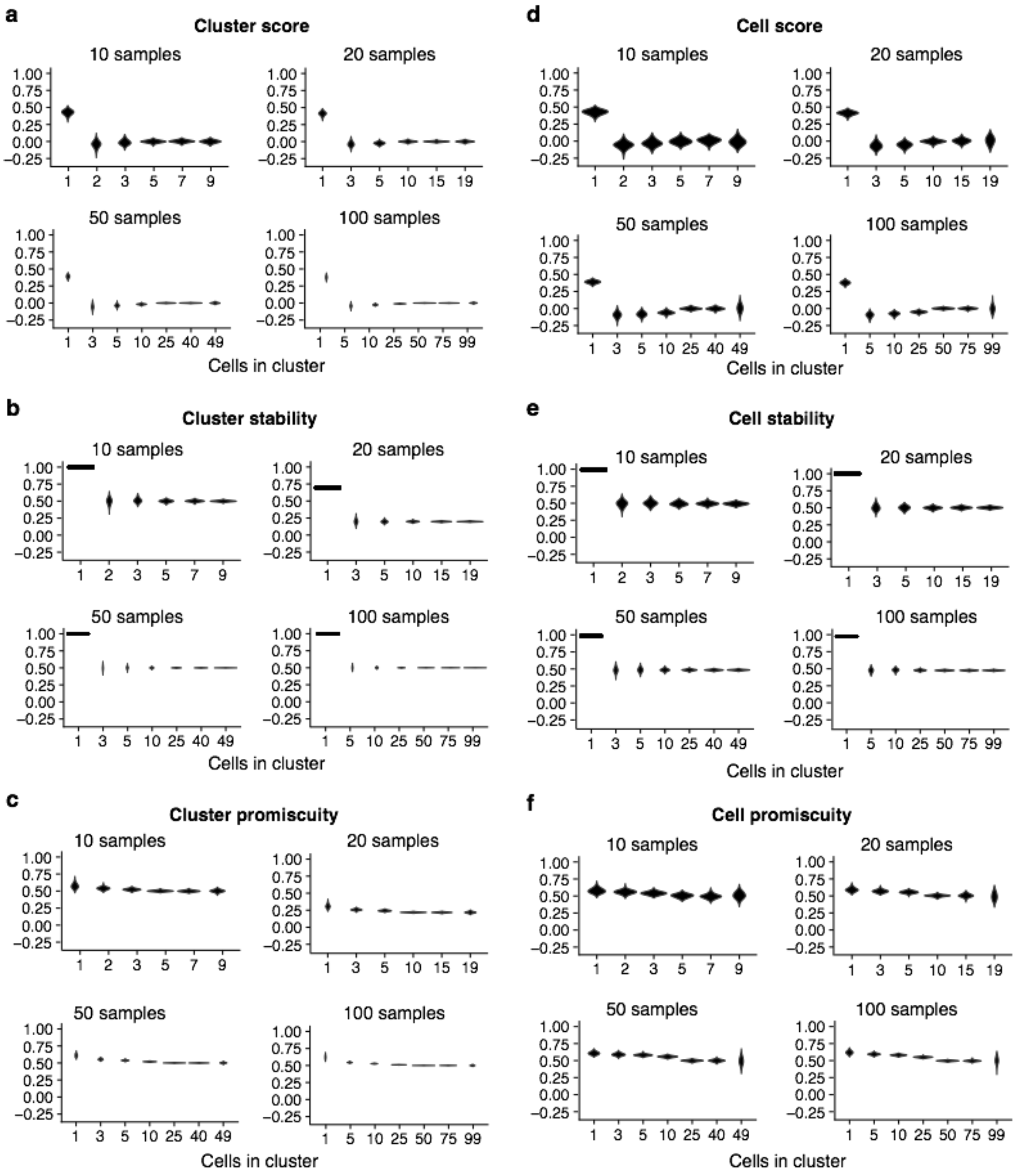

Supplementary Figure 1 Distributions illustrating the expected values of BEARscc metrics for completely random clusters of variable size. Violin plots display the cluster (**a-c**) and cell (**d-f)** score (**a,d**), stability (**b, e**), and promiscuity (**c, f**) computed from consensus matrices in which every cell is equally likely to associate with any other cell.

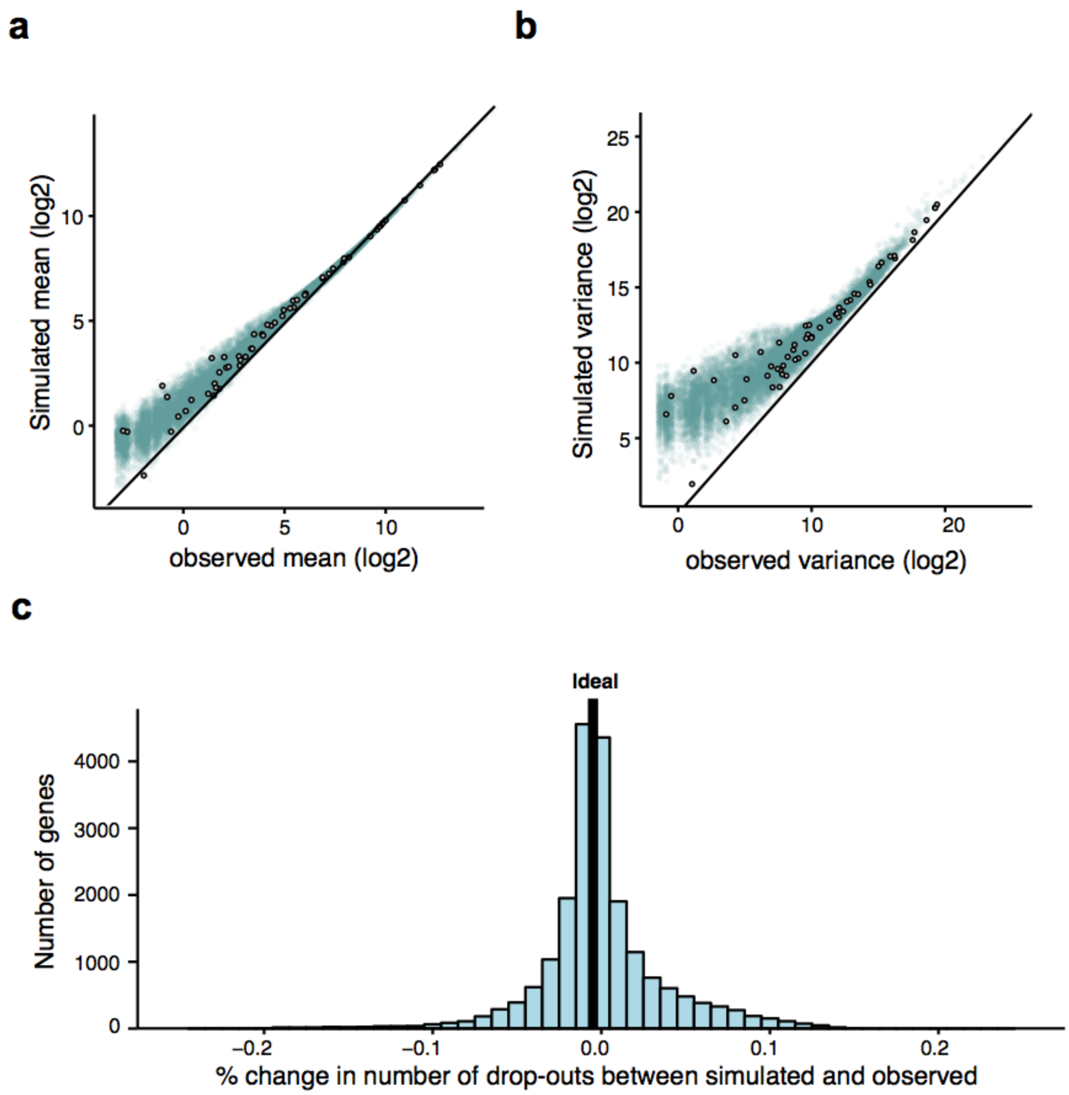

Supplementary Figure 2 BEARscc accurately models technical variability. Scatterplots of observed vs simulated mean expression **(a)** and variance in expression **(b)**, based on data from brain RNA control experiment. ERCC spike-in values are circled in black, human genes are shown in blue. **c**, Difference between simulated and observed drop-out frequency across genes.

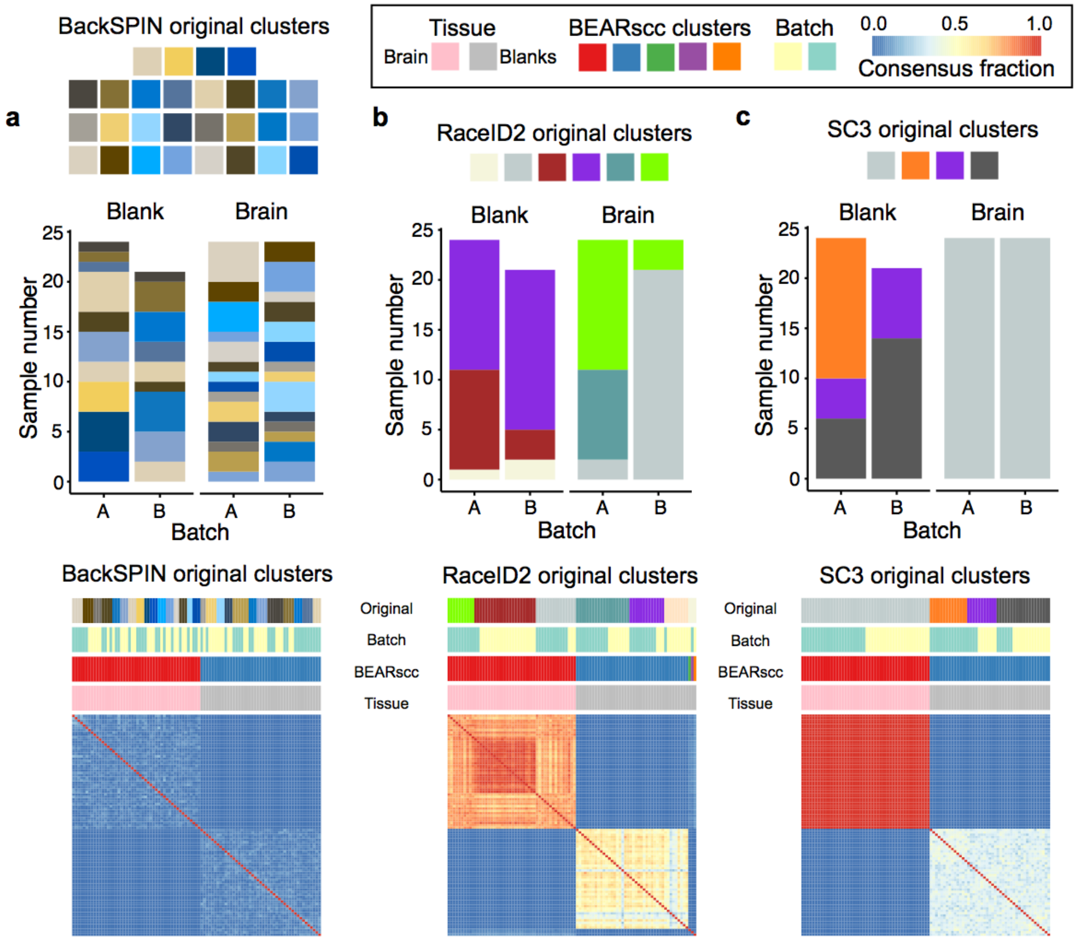

Supplementary Figure 3 BEARscc applied to the brain-and-blanks control experiment in combination with BackSPIN **(a)**, RaceID2 **(b)** and SC3 **(c)**. Top: bar graphs showing how the clusters generated by using each clustering algorithm alone ('original clusters') relate to sample type (brain or blank) and batch (A or B). RaceID2 and SC3 clusters are visibly confounded by batch. Bottom: for BEARscc applied with each algorithm, the noise consensus matrix is shown. The bars above the matrix show (from top): original clusters with algorithm alone, the batch, clusters derived after application of BEARscc, and the sample type.

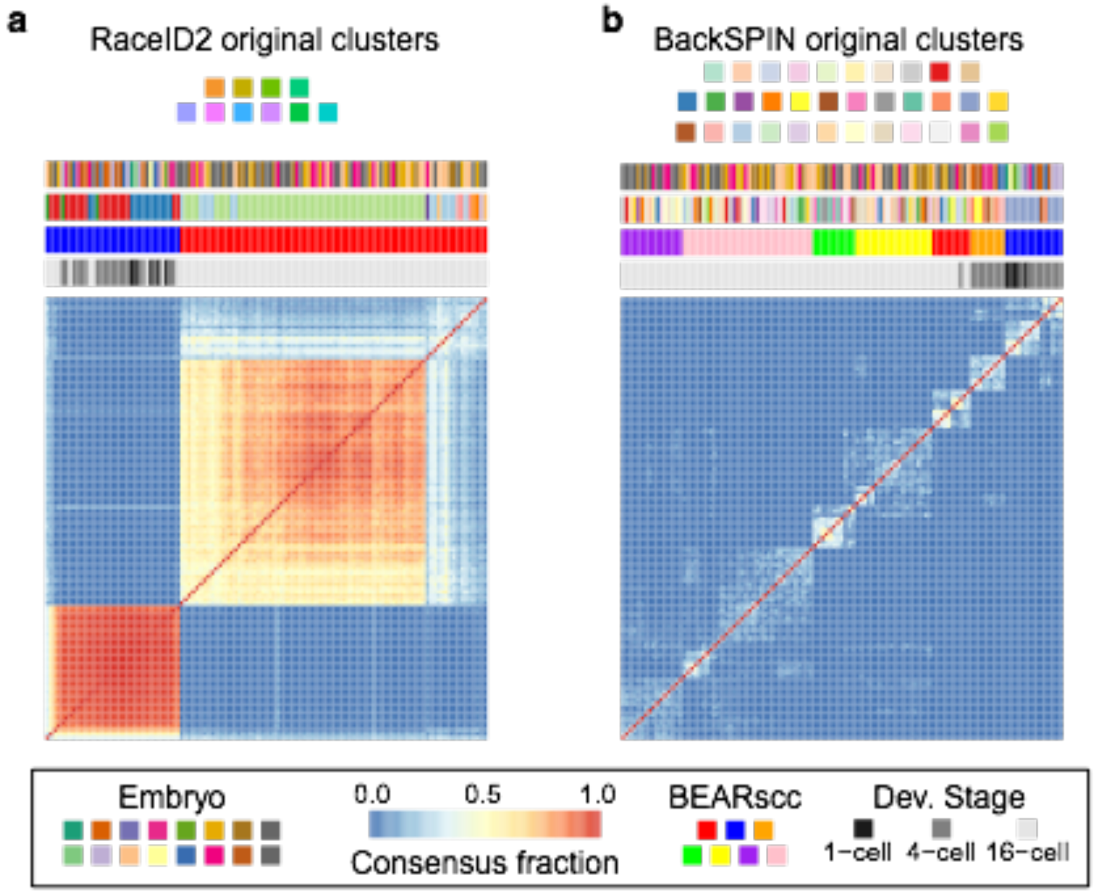

Supplementary Figure 4 BEARscc applied to the 1, 4, and 16-cell stages of *C. elegans* single cells in combination with BackSPIN **(a)** and RaceID2 **(b)**. For BEARscc applied with each algorithm, the noise consensus matrix is shown. The bars above the matrix show (from top): original clusters with algorithm alone, embryo batch, clusters derived after application of BEARscc, and the sample type.

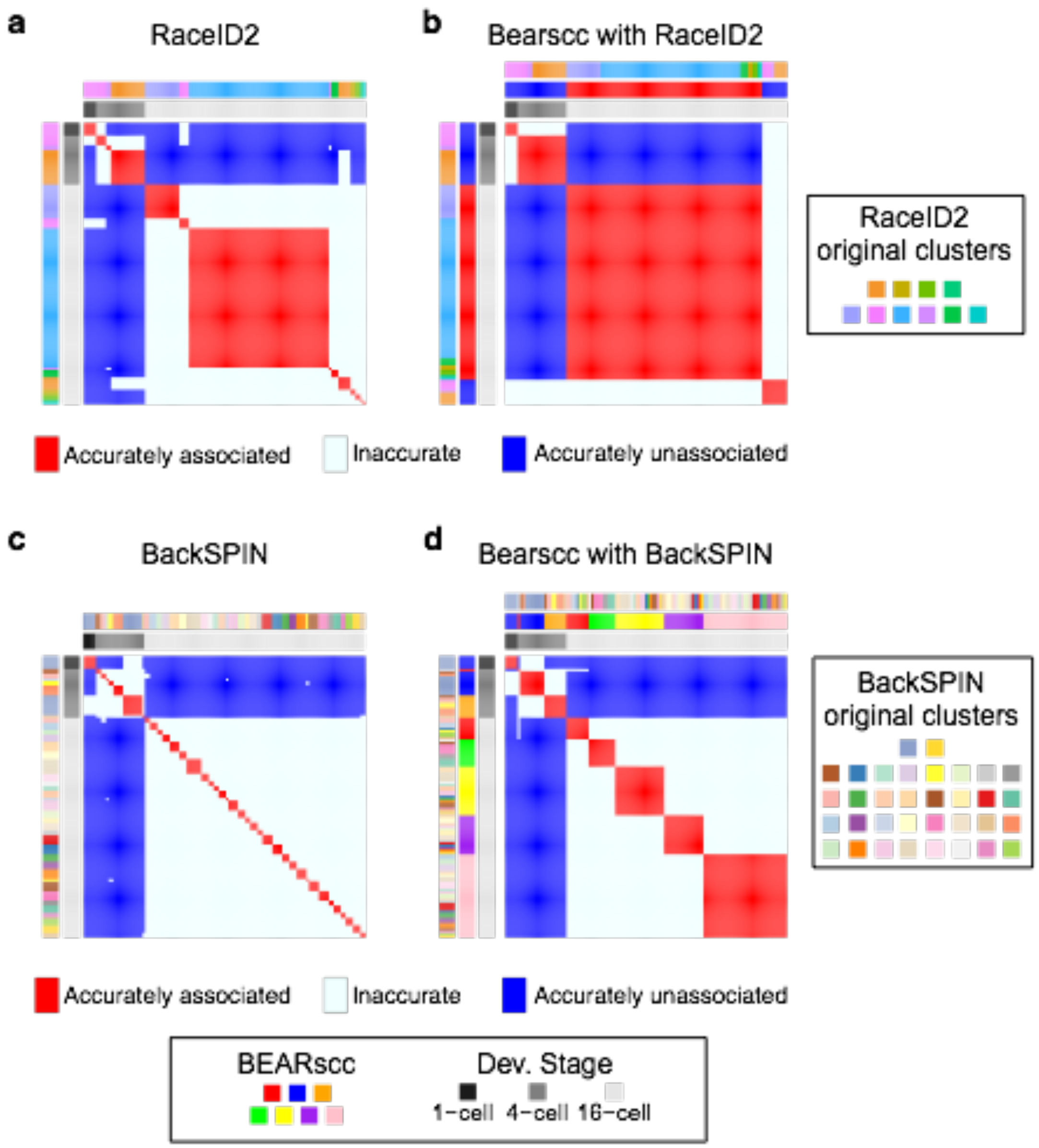

Supplementary Figure 5 Concordance between developmental stage and BEARscc applied to the 1, 4, and 16-cell stages of C. elegans single cells in combination with RaceID2 **(a-b)** and BackSPIN **(c-d)**. For RaceID2 alone **(a)**, BEARscc applied to RaceID2 **(b)**, BackSPIN alone **(c)**, and BEARscc applied to BackSPIN each algorithm **(d),** a concordance matrix indicates whether cell associations in the developmental stage and in the respective method classification agree and are present (red) or absent (blue) , or do not agree (light blue). The bars above the matrices show (from top): original clusters with algorithm alone, embryo batch, clusters derived after application of BEARscc, and the sample type (developmental stage).

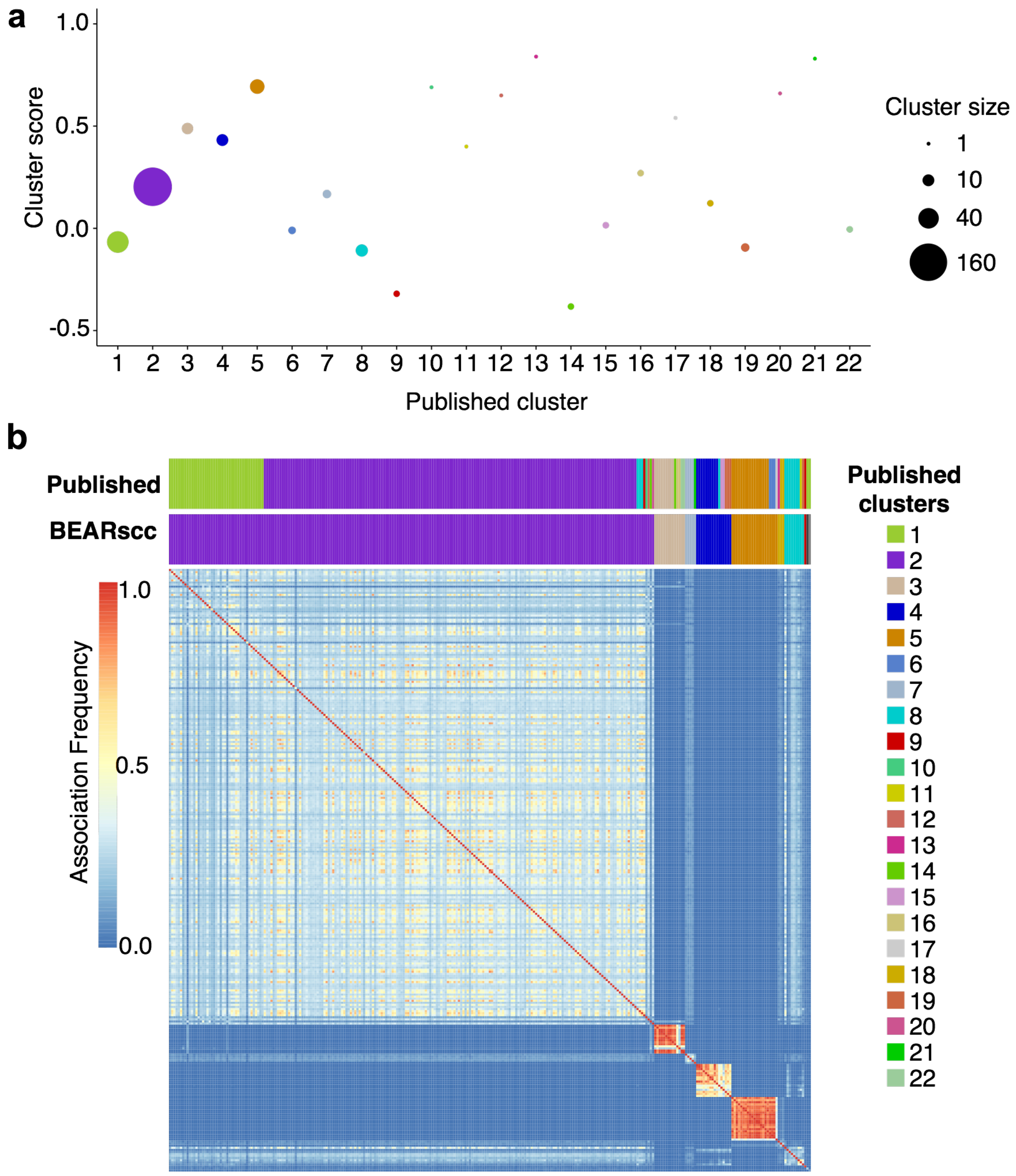

Supplementary Figure 6 BEARscc identifies robust clusters in data from murine intestinal cells. **a**, Cluster scores for "main" clusters (1-5) and outlier clusters (6-22). Circle size reflects number of cells per cluster. Colors are the same as in subfigure b. **b**, BEARscc noise consensus matrix for murine intestinal cells clustered with RaceID2. Above heatmap: published clusters (top) and noise consensus clustering (bottom, colors indicate closest match in the published clustering).

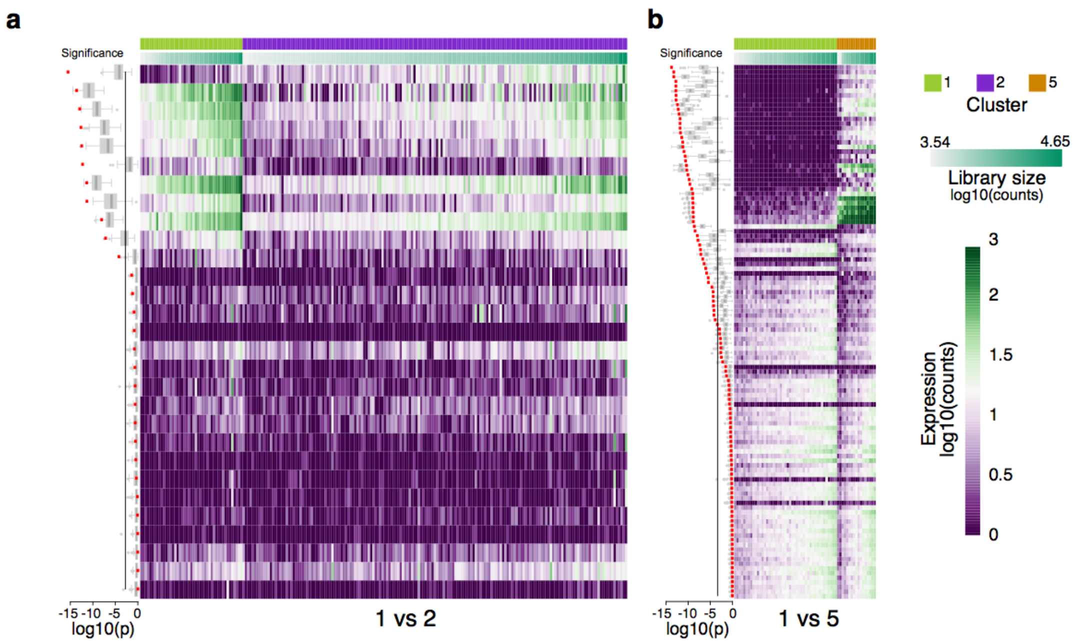

Supplementary Figure 7 BEARscc correctly detects that separation of "stem-like" cell clusters 1 and 2 is based on weak expression differences. (**a**) Heatmap of expression of genes characteristic of clusters 1 and 2 (as described in the original manuscript), and (**b**) clusters 1 and 5. Columns in each heatmap are ordered by library size per cell, rows sorted by significance of expression fold-change between clusters. Boxplots on the left denote the significance of difference in expression between the two clusters (Wilcoxon rank-sum test). Red denotes the observed values, and simulated technical replicates are shown in gray. Black solid vertical line denotes Bonferroni-corrected significance threshold.

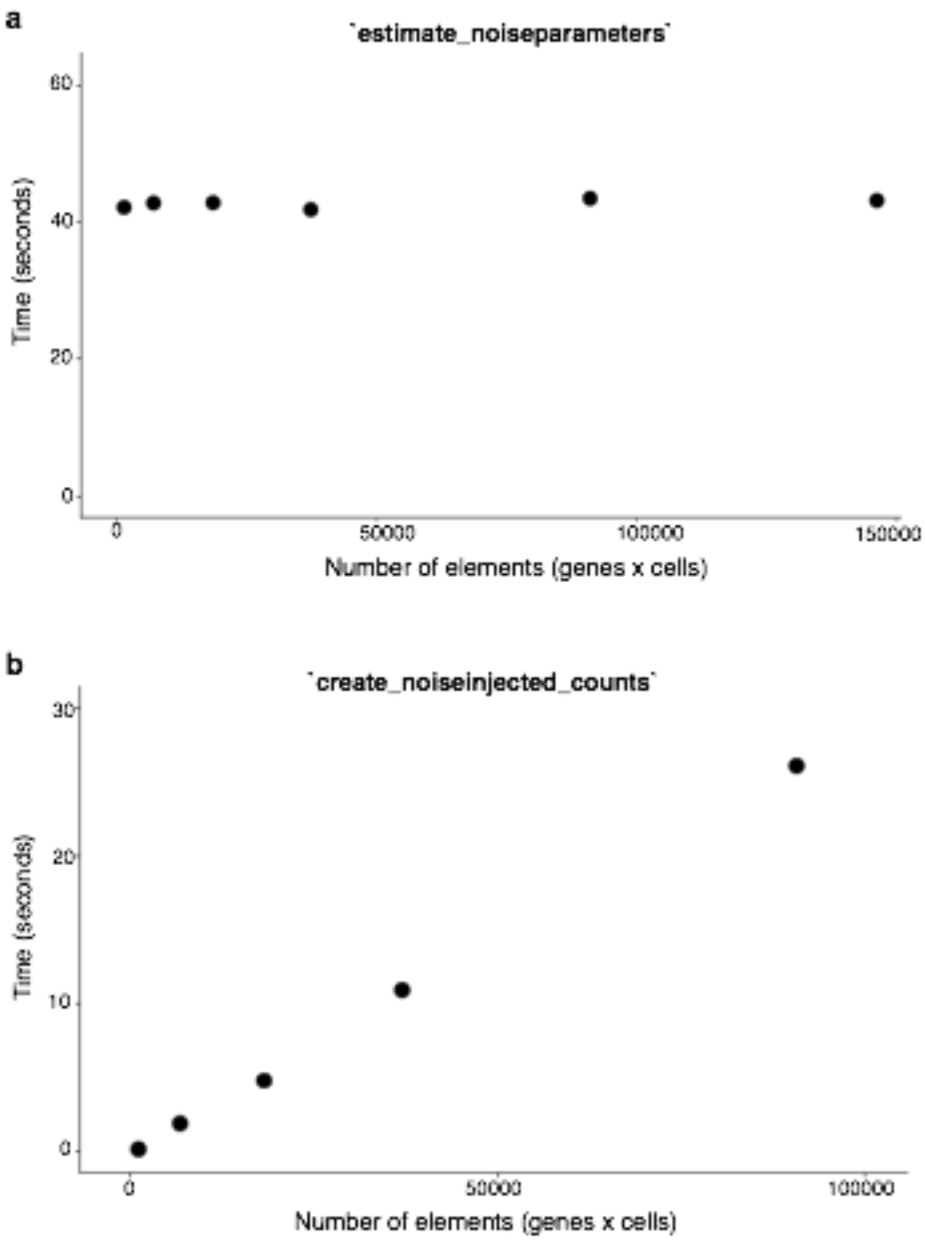

Supplementary Figure 8 BEARscc scales with time complexity O(number of cells x number of genes). Scatterplots display the relationship between the number of cells and genes and the run-time of the two time-critical functions `estimate_noiseparameters` (**a**) and `simulate_replicates` (**b**). Simulating replicates is the rate-limiting step, with run-time increasing linearly with the number of genes/cells in the experiment.
